# Launching an independent research laboratory in a digital era: practical lessons for early career investigators

**DOI:** 10.1038/s44277-026-00061-4

**Published:** 2026-04-23

**Authors:** Christine A. Rabinak

**Affiliations:** Department of Pharmacy Practice, Wayne State University, Detroit, MI USA

**Keywords:** Research management, Careers

## Abstract

The transition from trainee to principal investigator represents a major inflection point in an academic research career. While doctoral and postdoctoral training programs provide extensive preparation in experimental design, data analysis, and scientific communication, far less attention is devoted to the practical responsibilities associated with launching and managing an independent research laboratory. Early career investigators must rapidly develop skills in leadership, personnel management, infrastructure planning, and strategic funding development while simultaneously establishing their scientific identity. These challenges have intensified in the modern digital era, as research programs increasingly rely on sophisticated data management systems, collaborative technologies, and interdisciplinary networks. In this Perspective, I discuss several practical considerations that shape the early development of independent research laboratories, including research program design, startup planning, team leadership, collaborative partnerships, and strategic funding trajectories. By highlighting common challenges and lessons learned during the transition to independence, this article aims to help demystify the process of launching a research laboratory and to support early career investigators as they build sustainable scientific programs.

## Lay Summary

Starting a research lab is a major career transition, but scientists often receive little training in how to manage teams, resources, and collaborations. This article shares practical insights on building a successful lab, including planning research, leading teams, and navigating funding in a digital and collaborative environment. By making these often-hidden challenges more visible, the work aims to better prepare early career scientists for independence and long-term success.

## The transition to research independence

For many scientists, launching an independent research laboratory is often viewed as a defining milestone that follows years of graduate and postdoctoral training. Yet the transition to independence often reveals a reality that differs substantially from expectations. Investigators who were previously focused on experiments and publications suddenly become responsible for building teams, managing personnel, coordinating collaborations, and establishing the infrastructure needed to sustain a research program. In effect, launching a laboratory requires scientists to transition from individual contributors to leaders of small research organizations.

Despite the central importance of these responsibilities, most scientific training programs devote relatively little attention to the practical aspects of running a research group. Graduate and postdoctoral training typically emphasize experimental design, data analysis, and scientific communication. Far less time is spent discussing how to hire and mentor personnel, develop laboratory systems, manage collaborations, or design a long-term research trajectory. The early years of independence therefore often involve a steep learning curve as investigators develop the leadership and organizational skills necessary to sustain a research program.

The responsibilities associated with launching a laboratory have also evolved as the scientific landscape has become increasingly digital and collaborative. Contemporary research programs depend on complex data management systems, collaborative technologies, and computational tools. Scientific discovery increasingly occurs within interdisciplinary teams that may span institutions and geographic regions. These developments require investigators to manage data workflows, coordinate collaborations, and navigate issues related to authorship and data sharing.

**Fig. 1:**
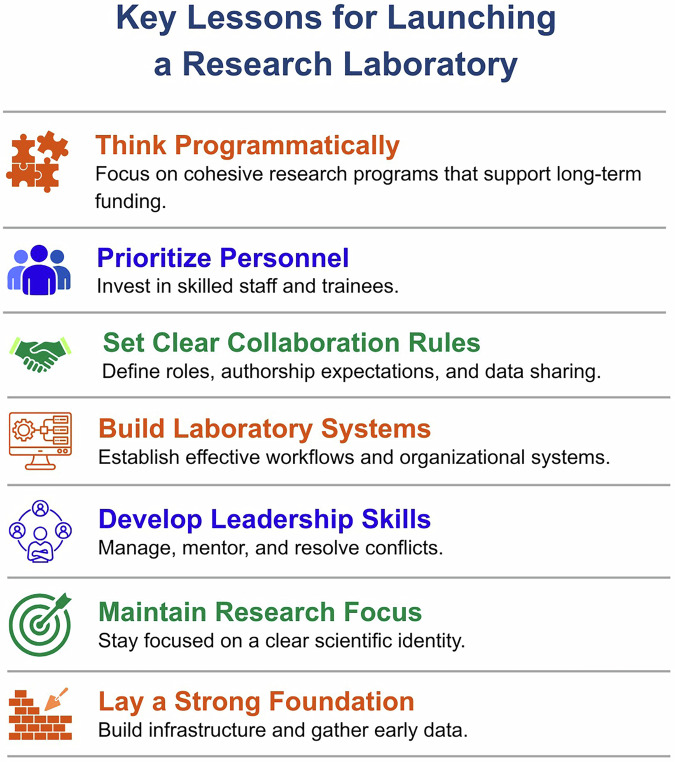
Key Lessons for Launching a Research Laboratory. A summary of core principles that support the successful establishment of an independent research program. Key lessons include developing a cohesive scientific vision, investing in personnel, establishing clear collaboration practices, building laboratory systems early, strengthening leadership and management skills, maintaining a focused research identity, and laying the foundational infrastructure required for long‑term productivity and funding success.

For early career investigators, the first several years of independence are therefore not solely about producing scientific discoveries. They are also about building the intellectual and organizational foundations that will sustain a research program over time. Decisions made during these early years regarding research focus, personnel, infrastructure, and collaborations often shape the trajectory of a laboratory for many years. Across these experiences, several recurring lessons emerge that consistently shape the success of newly established laboratories (Fig. 1). This Perspective is intended for scientists spanning the late postdoctoral period through the first several years of independence. Its goal is not to provide prescriptive instructions, but rather to translate commonly encountered challenges during this transition into concrete, implementable leadership and organizational practices that early career investigators can adapt to their own research environments.

## Designing a research program

One of the most consequential early decisions for a new principal investigator is defining the scientific vision that will guide the laboratory’s research program. During graduate and postdoctoral training, researchers often contribute to projects designed by mentors or collaborative teams. Transitioning to independence requires investigators to articulate a distinct intellectual identity and to develop a program of work capable of sustaining both scientific discovery and long-term funding. Successful laboratories are rarely built around isolated studies. Instead, they typically develop around a cohesive set of scientific questions that evolve as new findings emerge. These questions must be sufficiently focused to establish a recognizable area of expertise while remaining broad enough to support multiple projects, trainees, and funding opportunities. Establishing such a research vision is widely recognized as a critical step for investigators transitioning to independence [1].

Designing a research program begins by identifying how prior training and methodological expertise can be applied to emerging problems. Early projects establish workflows, generate pilot data, and produce initial independent publications. Working backward from long-term funding goals can help clarify how early projects should be structured. If an investigator aims to submit a major external grant within several years of launching a laboratory, the pilot data, methodological expertise, and collaborative partnerships needed for that application can be mapped in advance. To translate these principles into practice, many investigators operationalize this approach by articulating a brief research mission statement and mapping early projects onto a 3–5‑year funding trajectory. For example, pilot studies are often selected not only for scientific interest, but for their ability to generate preliminary data, establish workflows, and support early career funding mechanisms aligned with longer‑term grant goals.

The design of research programs has also been shaped by the increasing role of digital infrastructure in modern science. Laboratories now rely on specialized software for data analysis, collaborative platforms for project management, and secure systems for storing and sharing research data. Early consideration of these elements can help ensure that research workflows remain organized, scalable, and reproducible as laboratories grow.

## Startup planning and infrastructure

Launching a laboratory requires careful consideration of the infrastructure needed to support the planned research program. Discussions about startup packages often emphasize equipment purchases, yet the early success of a laboratory rarely depends on equipment alone. In practice, effective startup planning involves building an operational ecosystem that includes personnel, institutional infrastructure, and systems that support data collection and analysis.

In many laboratories, personnel represent the most important early investment. Experienced research staff, coordinators, or postdoctoral fellows often play a critical role in establishing workflows, managing studies, and maintaining continuity across projects. These individuals help transform a laboratory from a conceptual plan into a functioning research environment capable of generating pilot data and supporting grant applications. Operational costs are often underestimated. Beyond equipment and salaries, laboratories must budget for participant compensation, software licenses, data storage, transcription services, and core facility fees. These costs can substantially affect the financial runway before external funding is secured.

The infrastructure supporting modern research laboratories is also increasingly shaped by digital technologies. Secure data storage, collaborative writing platforms, and project management systems now form the backbone of many research programs. Effective data management practices that span the research lifecycle are essential for maintaining transparency, reproducibility, and long-term accessibility of research data [2, 3]. The goal of digital infrastructure selection is not to identify a single “best” platform or to adopt every available technology. Rather, it is to choose systems that align with a laboratory’s regulatory requirements, research workflows, and scale of activity. Across domains, in practice, successful laboratories emphasize consistency, clear access permissions, and shared norms for use. Importantly, early adoption of even simple systems, when paired with shared expectations, can substantially reduce cognitive and administrative load as laboratories scale. Table 1 highlights representative categories of digital infrastructure commonly used in research laboratories, along with considerations that may inform selection based on local needs.

**Table 1:** Core Digital Infrastructure for Research Laboratories: Functions, Examples, and Key Considerations.

Function	Example Tools	Key Considerations
**Data storage and backup**	Institutional servers; secure academic cloud storage (e.g., Box, OneDrive, Google Drive via institution); commercial cloud storage (e.g., AWS S3); encrypted external drives; discipline-specific repositories (e.g., OSF)	Ensure compliance with regulatory requirements (e.g., HIPAA, IRB), robust access controls, automatic backup, version history, and long-term data stability. Institutional systems are often compliant and secure but may be less flexible; cloud platforms are scalable and user-friendly but require careful permission management.
**Electronic lab notebooks (ELNs)**	Benchling, LabArchives, Open Science Framework (OSF), eLabFTW, Notion-based systems	Prioritize searchability, version history, audit trails, and integration with data workflows. Formal ELNs support reproducibility and compliance but may involve licensing costs; flexible systems require clearly defined lab norms and onboarding for consistent use.
**Project and task management**	Trello, Asana, Notion, Monday.com, ClickUp	Effective systems support task assignment, timeline tracking, and visibility across projects. Simpler tools improve adoption; more complex platforms support multi-project coordination but require onboarding and maintenance.
**Collaborative writing and document management**	Google Workspace; Microsoft Teams/SharePoint; Overleaf (LaTeX)	Real-time collaboration, version control, and commenting are critical. Platform choice may be influenced by institutional subscriptions and security policies.
**Version control and code management**	GitHub; GitLab; Bitbucket	Supports reproducibility, transparency, and collaborative development. Private repositories are often necessary for sensitive or unpublished work. Adoption may require training and clear documentation practices.
**Scheduling and resource coordination**	Google Calendar; Outlook; Calendly; Doodle; Skedda (equipment booking)	Helps coordinate meetings, participant scheduling, and shared resources. Automated scheduling tools reduce administrative burden but should align with institutional workflows.
**Internal communication**	Slack; Microsoft Teams; email listservs	Supports real-time coordination and information sharing. Clear communication norms and intentional channel use are essential to prevent overload and fragmentation across platforms.
**Data organization and documentation**	Standardized folder structures; README files; data dictionaries	Not tool-specific but essential for reproducibility. Consistent naming conventions, metadata, and documentation practices support long-term data usability and team coordination.
**Data analysis and visualization environments**	R/RStudio, Python/Jupyter notebooks, SPSS, SAS	Selection is often guided by disciplinary norms, reproducibility requirements, and trainee expertise. Script-based environments support transparency and reproducibility but may require greater initial training investment.
**Secure data transfer and sharing**	Box Secure Share; OneDrive secure links; Globus; institutional file transfer systems	Critical for multi-site collaborations and sensitive data. Ensure encryption, access tracking, and compliance with institutional and funding requirements.

Examples are intended to be illustrative rather than exhaustive. Final tool selection should be guided by institutional policies, regulatory requirements, disciplinary norms, and the scale and complexity of the research program.

Ultimately, startup resources should be viewed not simply as a list of purchases but as the strategic construction of a laboratory’s operational foundation. Careful allocation of these resources, particularly toward personnel and digital infrastructure, can accelerate the generation of preliminary data and position investigators for competitive grant applications.

## Building and leading a research team

One of the most significant changes accompanying the transition to research independence is the responsibility for hiring and leading a research team. During training, scientists typically work within research environments where responsibility for personnel management rests with senior investigators. Upon launching an independent laboratory, however, investigators become responsible for shaping the laboratory’s culture, expectations, and working environment. Graduate students and postdoctoral fellows may benefit from opportunities to mentor others, participate in hiring, and contribute to budget development and grant writing. These experiences can provide early exposure to the leadership and organizational tasks that become central during the transition to running an independent laboratory.

Hiring decisions are particularly consequential during the early stages of a laboratory. Initial staff members often help establish research workflows, develop procedures, and train new personnel as the group expands. For this reason, hiring decisions should consider not only technical expertise but also reliability, communication skills, and alignment with the laboratory’s culture. Reference checks can provide valuable insight into these qualities and may reveal information that is not readily apparent during interviews.

Leading a research team also requires investigators to develop management and leadership skills that are rarely emphasized during traditional scientific training. Effective mentorship, clear communication, and supportive research environments have been identified as important factors shaping the development of early career researchers [4]. Managing personnel involves setting expectations, providing constructive feedback, and fostering an environment in which trainees feel supported in their scientific development. Laboratory culture plays a central role in shaping both productivity and trainee experiences. Research groups that emphasize transparency, accountability, and mutual respect are more likely to foster environments in which team members feel comfortable discussing challenges and contributing ideas. Intentional leadership practices can therefore help laboratories align scientific productivity with the development of inclusive and supportive research environments [1]. In practice, concrete practices that support effective team leadership often include brief onboarding documents defining roles and expectations, regular one‑on‑one meetings to provide feedback, and clearly articulated authorship or project ownership policies. Although these tools are often informal, they can substantially reduce ambiguity and support productive working relationships as laboratories grow.

## Collaborations in a networked scientific environment

Scientific discovery increasingly occurs within collaborative networks that span disciplines and institutions [5, 6]. Research teams have become the dominant mode of knowledge production across most scientific fields [7]. For investigators launching new laboratories, collaborations can therefore play an important role in expanding the scope and impact of research programs.

Collaborative partnerships may provide access to complementary expertise, specialized methodologies, or participant populations that would be difficult to obtain within a single laboratory. Interdisciplinary collaborations are particularly valuable as research questions become more complex and require integration of diverse approaches. At the same time, collaborations introduce challenges that new investigators must learn to navigate. Questions related to authorship, intellectual contributions, data ownership, and project leadership can become sources of tension if expectations are not clearly established early in the collaboration. Early career investigators may be particularly vulnerable to ambiguity in collaborative roles, especially when working within large multi-investigator initiatives.

Many investigators address these challenges by establishing expectations early through brief written summaries that outline roles, timelines, data access, and authorship criteria. These documents are typically designed to support shared understanding rather than formal enforcement. Even informal documentation can reduce misunderstandings as projects evolve. Digital communication tools and collaborative platforms have made it easier for distributed teams to coordinate research activities, but they also require clear systems for communication and data organization. When managed thoughtfully, collaborations can substantially expand the intellectual reach of a research program while strengthening the networks that support scientific discovery.

## Strategic planning for research funding and sustainability

Establishing a sustainable research program requires investigators to think strategically about funding trajectories from the earliest stages of independence. While trainees often focus on discrete research projects, principal investigators must consider how individual studies contribute to a broader research agenda capable of supporting multiple funding opportunities over time. Working backward from long-term funding goals can help clarify how early projects should be structured. Pilot studies often serve as building blocks that establish proof of concept, generate preliminary data, and support future grant applications. Early projects contribute to both scientific discovery and the evidence base for sustained funding. Early career investigators should also take advantage of funding mechanisms designed specifically to support the transition to independence. Federal agencies and private foundations frequently offer grants targeted to investigators in the early stages of their careers. Because eligibility for these programs is often time limited, identifying and pursuing these opportunities early can be an important component of establishing a stable funding trajectory.

**Fig. 2:**
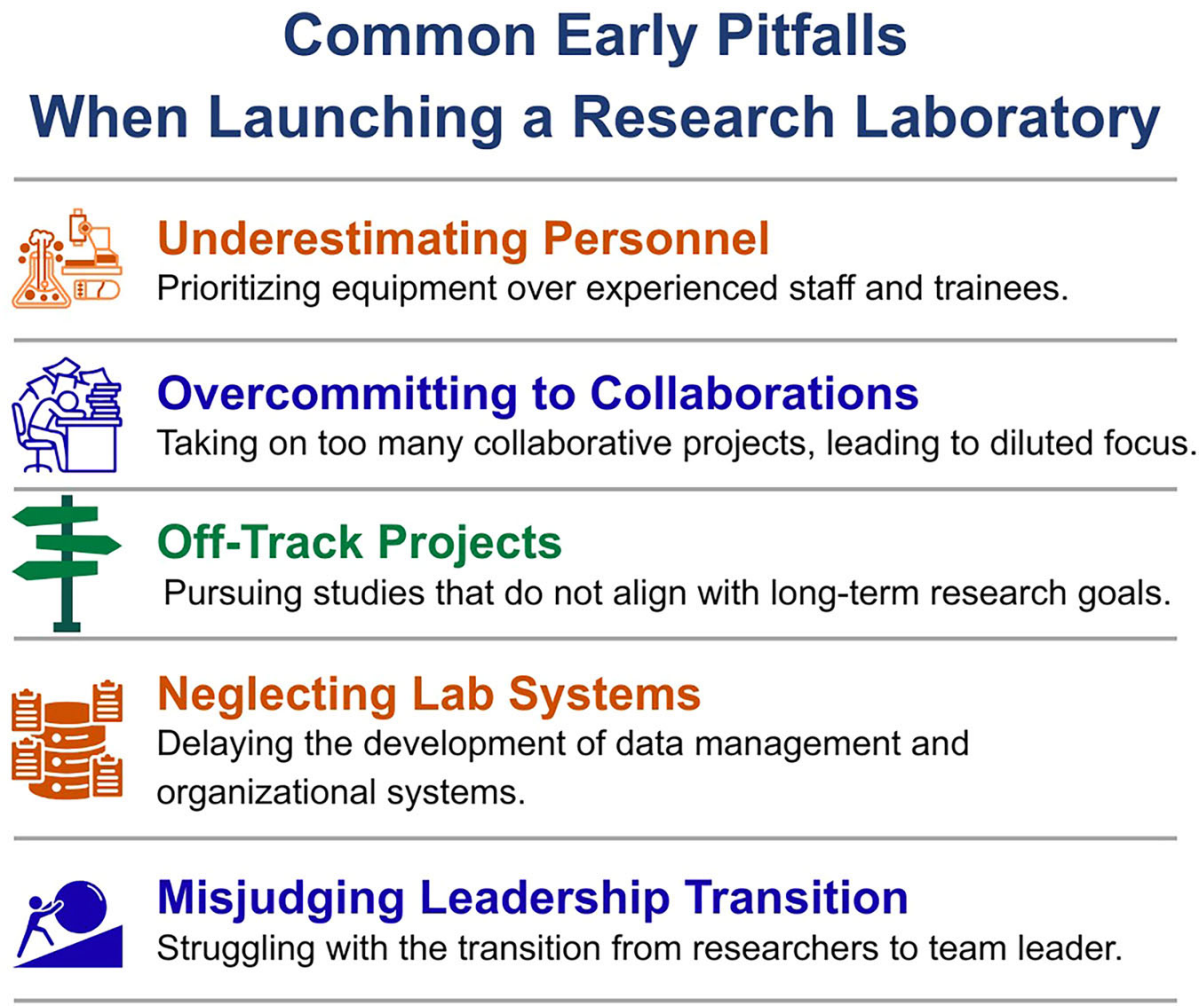
Common Early Pitfalls When Launching a Research Laboratory. An overview of common challenges encountered during the first years of independence. Frequent pitfalls include underestimating the importance of personnel, overcommitting to collaborations, pursuing projects that lack alignment with long‑term research goals, delaying the development of laboratory systems, and underappreciating the leadership transition from trainee to principal investigator.

Maintaining coherence within a research program is equally important. Although new investigators may encounter numerous collaborative opportunities, excessive diversification during the early years of independence can dilute the development of a clear scientific identity. Maintaining a focused trajectory helps investigators build a coherent scientific identity while avoiding several common pitfalls that frequently emerge during the early stages of independence (Fig. 2). In practice, coherence is often maintained through periodic reassessment of how new opportunities align with the laboratory’s central research questions and funding strategy. Some investigators find it useful to revisit these priorities annually, using them as a lens for deciding which collaborations, service activities, or new projects to pursue or defer.

## The first three years of independence

**Fig. 3:**
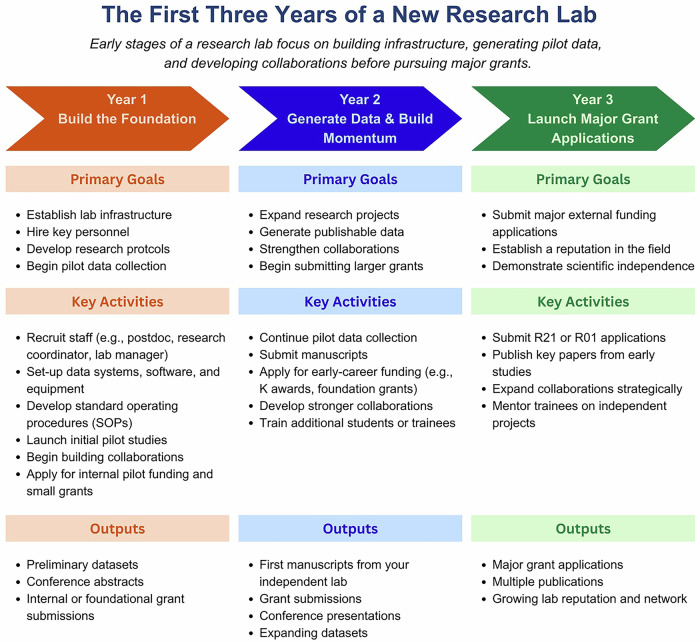
The First Three Years of a New Research Lab: A Realistic Roadmap. A timeline outlining typical goals, activities, and outputs during the initial three years of launching a research laboratory. Year 1 focuses on establishing infrastructure, hiring personnel, developing protocols, and generating preliminary data. Year 2 emphasizes data production, manuscript submissions, growing collaborations, and early‑career grant applications. Year 3 centers on submitting major grant applications, publishing key studies, mentoring trainees, and demonstrating scientific independence.

Although each laboratory develops differently, many research groups follow a similar developmental pattern during the first several years of independence (Fig. 3). These early years are often characterized by uncertainty, rapid learning, and recalibration of expectations.

During the first year of independence, much of the investigator’s effort is devoted to establishing the operational structure of the laboratory. This period often includes hiring key personnel, developing protocols, obtaining regulatory approvals, and initiating pilot studies. To support reproducible workflows, some laboratories also implement structured “data management” practices, such as periodic lab‑wide organization sessions during which datasets are backed up, file structures are reviewed, and project timelines are updated. These routines can help normalize good data practices and reduce downstream challenges as research activity scales. Although these activities are essential for launching a research program, they may not immediately produce traditional academic outputs such as publications or major grant submissions.

By the second year, research activity often begins to gain momentum. Laboratory personnel become more proficient with established procedures, data collection pipelines become more reliable, and early studies begin to generate analyzable datasets. These findings frequently support conference presentations, preliminary manuscripts, and early grant submissions. By the third year, many laboratories begin transitioning toward sustained scientific productivity. Pilot data generated during the earlier stages can support more competitive grant applications, and the first publications from the laboratory’s independent research program may begin to appear. Although the trajectory of a laboratory rarely follows a perfectly linear path, investments made during these early years frequently determine the long-term direction of a research program.

## Preparing the next generation of research leaders

The transition from trainee to principal investigator represents a pivotal stage in the scientific career pathway. While doctoral and postdoctoral training provide extensive preparation in research methods and scientific communication, far less attention is devoted to the practical responsibilities associated with launching and managing a research laboratory. As a result, many investigators develop leadership and management skills largely through experience. Supporting early career investigators requires greater transparency about research leadership. Mentorship, leadership training programs, and institutional resources can play important roles in helping investigators develop the skills required to manage teams, coordinate collaborations, and sustain research programs. In addition to formal mentorship, virtual peer networks have emerged as important sources of support for investigators navigating early independence. Online communities such as New PI Slack and Future PI Slack provide forums for sharing experiences, troubleshooting challenges, and accessing collective knowledge related to hiring, lab management, and funding strategies.

Ultimately, building a successful laboratory requires more than scientific expertise alone. It involves building the people, systems, and collaborative environments that allow discovery to occur. Practical planning tools related to these themes have been compiled into an openly accessible toolkit for early career investigators [8]. Additional, more in‑depth resources focused specifically on the human and organizational dimensions of lab leadership, including hiring, mentorship, culture, and performance management, have also been developed to support investigators seeking more structured guidance in these areas [9]. By sharing lessons learned during the transition to independence, the scientific community can help demystify the process of launching a research laboratory and better prepare the next generation of research leaders. Greater transparency may ultimately strengthen the scientific enterprise by enabling investigators to focus on discovery.

### Citation diversity statement

The authors have attested that they made efforts to be mindful of diversity in selecting the citations used in this article.
